# Exploring Inflammatory Status in Febrile Seizures Associated with Urinary Tract Infections: A Two-Step Cluster Approach

**DOI:** 10.3390/brainsci11091168

**Published:** 2021-09-03

**Authors:** Raluca Maria Costea, Ionela Maniu, Luminita Dobrota, Rubén Pérez-Elvira, Maria Agudo, Javier Oltra-Cucarella, Andrei Dragomir, Ciprian Bacilă, Adela Banciu, Daniel Dumitru Banciu, Călin Remus Cipăian, Roxana Crișan, Bogdan Neamtu

**Affiliations:** 1 Pediatric Research Department, Pediatric Clinical Hospital Sibiu, 550166 Sibiu, Romania; ionela.maniu@ulbsibiu.ro; 2 Pediatric Neurology Department, Pediatric Clinical Hospital Sibiu, 550166 Sibiu, Romania; 3 Faculty of Medicine, Lucian Blaga University of Sibiu, 550024 Sibiu, Romania; luminitadobrota@yahoo.com (L.D.); ciprian.bacila@ulbsibiu.ro (C.B.); calin.cipaian@ulbsibiu.ro (C.R.C.); crisanrox@gmail.com (R.C.); 4 Research Center in Informatics and Information Technology, Mathematics and Informatics Department, Faculty of Sciences, Lucian Blaga University of Sibiu, 550024 Sibiu, Romania; 5 Neuropsychophysiology Laboratory, NEPSA Rehabilitación Neurológica, 37003 Salamanca, Spain; rubenperezelvira@gmail.com (R.P.-E.); mjagudojuan@gmail.com (M.A.); 6 Department of Health Psychology, Universidad Miguel Hernández de Elche, 03202 Elche, Spain; joltra@umh.es; 7 N.1 Institute for Health, National University of Singapore, Singapore 117575, Singapore; andrei.drag@gmail.com; 8 Department of Bioengineering and Biotechnology, Faculty of Medical Engineering, Politechnic University of Bucharest, 011061 Bucharest, Romania; adela.banciu79@gmail.com (A.B.); danieldumitrubanciu@gmail.com (D.D.B.); 9 Computer and Electrical Engineering Department, Faculty of Engineering, Lucian Blaga University of Sibiu, 550024 Sibiu, Romania

**Keywords:** febrile seizures, urinary tract infections, inflammatory biomarkers, cluster analysis, laboratory data, CRP, cut-off values, urine leukocyte and nitrite stick test

## Abstract

Background: Urinary tract infections (UTIs) are considered common facilitating factors, along with other infections, in triggering febrile seizures (FS). The main purpose of our study was to identify specific inflammatory patterns of UTI cases from other infections in a specific cluster, using a combination of inflammatory biomarkers to differentiate UTIs from other bacterial diseases triggering FS. Method: This prospective study included a number of 136 patients with 197 distinct FS events, from patients hospitalized in the Pediatric Clinical Hospital Sibiu, among which 10.2% were diagnosed with UTIs. Results: In one-third of the patients with UTIs (20 cases), the symptoms were limited to fever and FS. Using two-step cluster analysis, a distinct UTI inflammatory pattern has emerged: highest platelet values (PLT), median value 331 × 103/mm^3^ and intermediate C-reactive protein (CRP), median value 15 mg/dL, platelet distribution width (PDW), median value 9.65%, platelet-large cell ratio (P-LCR), median value 14.45%, mean platelet volume (MPV), median value 8.60 fL and neutrophil-to-lymphocyte values (NLR), median value 3.64. Furthermore, higher PDW (median value 12.25%), P-LCR (median value 28.55%), MPV (median value 10.40 fL), CRP (median value 74.00 mg/dL) and NLR values (median value 4.11) were associated mainly (85.7%) with bacterial lower respiratory infections. UTIs were highly unlikely in these patients with significantly increased CRP values and normal values of platelet indices. Conclusions: Considering the nonspecific clinical picture of UTIs at an early age, to optimize the management of FS, a fast diagnosis of UTI is mandatory. The analysis of the inflammatory biomarker clusters (rather than individual parameters) correlated with urine leukocyte and nitrite stick evaluation for specific age groups could help in identifying even oligosymptomatic UTIs patients. The study limitation (20 UTI cases) recommends future multicentric trials on larger datasets to validate the model.

## 1. Introduction

The International League Against Epilepsy (ILAE) has defined FS as seizures occurring in childhood after one month of age, associated with a febrile illness not caused by an infection of the central nervous system, without previous neonatal or unprovoked seizures, and not meeting criteria for other acute symptomatic seizures [1]. 

FS are the most common childhood neurological disorders and an important health problem with potential short and long-term complications [2]. The benign nature of FS should be carefully reconsidered due to variable outcomes associated with atypical presentations. Respiratory tract infections, predominantly viral, are the most common childhood infectious diseases worldwide and the main fever triggers in FS. The genetic predisposition increases the risk of FS. Furthermore, it seems that children with FS have an augmented pro-inflammatory response of (TNF-α), interleukin-6 (IL-6), and interleukin 1-β (IL-1β) released by macrophages. These pro-inflammatory cytokines seem to be unbalanced by the anti-inflammatory pairs, such as interleukin-1 receptor antagonist (IL-1Ra), IL-10 or by the improper acetylcholine secretion to inhibit the release of TNF-α, IL-1β, and IL-18 from macrophages [3,4]. Bacterial infections are also important triggers because of the lipopolysaccharide (LPS) or lipoteichoic acid (LTA) component in the cell wall of Gram-negative or Gram-positive bacteria (especially in Gram-negative bacteria). Consequently, the blood-brain barrier (BBB) permeability is altered, with subsequent elevated cytokines levels in the brain and neuroinflammation activation [4].

On the other hand, differences between the innate immune responses in viral and bacterial infections are acknowledged in the literature, therefore, the pro-inflammatory pattern triggering the febrile seizure event is different. A localized bacterial infection is characterized by specific molecular fingerprints of the incriminated pathogen. More details related to the inflammatory mechanism triggering the febrile seizure event and the differences in innate immune responses are presented in Appendix A. In this context, unsurprisingly, research reports suggest fundamental differences in innate immune responses to Gram-positive and Gram-negative bacteria due to the differences in cell wall architecture [5]. Therefore, it is crucial for a practitioner to classify the infection etiology and its localization, to properly address and manage the triggers for the seizure event. UTIs are considered common facilitating factors for the FS along with other bacterial infections.

The literature is scarce regarding the association between FS and localized infections such as UTIs. The nonspecific clinical picture of UTIs in childhood makes it difficult to differentiate from other bacterial infections triggering the FS events. In clinical practice, UTIs are diagnosed based on Kass criteria regarding the number of colonies forming units (CFU)/mL of sampled urine. The reference range for positive UTIs refers either to the presence of more than 10^4^ CFU/mL of urine and a clinical suggestive context or more than 10^5^ CFU/mL of urine. Along with urine cultures, leukocyte and nitrite stick evaluation, especially used in combination for specific age groups, could be a useful screening test with a good negative predictive value to rule out the UTIs [6,7].

The main goal of our study was to identify UTI cases, the specific patterns of upper UTIs and lower UTIs using a two-step cluster approach analysis. Our purpose was to find a combination of inflammatory biomarkers that differentiate UTIs from other bacterial diseases associated with FS. Cytokines involved in the pro-inflammatory responses are important, however higher costs and a narrow availability are essential disadvantages. Consequently, we focused on available, low-cost biomarkers, such as the peripheral blood NLR, MPV, and red cell distribution width (RDW) as indices for inflammation [3]. Recent reports highlight the importance of NLR, MPV, and RDW in severe systemic inflammation. They were correlated with inflammatory cytokines, erythrocyte sedimentation rate (ESR), CRP levels, and showed promise in differentiating simple from complex FS [3]. Moreover, machine learning algorithms might offer a solution to forecast a specific outcome (Appendix A).

## 2. Materials and Methods

### 2.1. Study Design

This prospective study was conducted between October 2013 and October 2016 at the Sibiu Pediatric Clinical Hospital as part of a larger project on febrile seizures [8,9]. The study protocol was approved by the Ethics Committee of the hospital and was carried out according to the Declaration of Helsinki principles, with the informed consent of the members. The study group included 136 patients with 197 different FS events. The inclusion criterion referred to all the children with a recent history (less than 24 h) of characteristic and unequivocal FS events regardless of the infectious etiology. We did not include in the study the following patients: (1) with a history of seizures without fever; (2) with central nervous system infections or other possible traumatic or metabolic causes of symptomatic acute seizures (dyselectrolytemia, hypoglycemia); (3) with uncertain or incomplete clinical data (according to International Epilepsy League revised principles-ILAE) [1]. We used the age criterion according to the revised definition of ILAE, referring to the age range between one month and five years. Simple febrile seizures were defined as generalized seizures lasting less than 15 min and without recurrence within 24 h. Complex febrile seizures were diagnosed based on the presence of at least one criterion from the following: focal appearance, duration over 15 min, and multiple seizures within 24 h [1].

### 2.2. Paraclinical Investigations in the Evaluation of the UTIs

Blood samples were drawn from the patients upon admission to the intensive care unit for the following workup: complete blood count, venous blood gases, random blood glucose, lactate, serum electrolytes, and CRP. CRP levels under 10 mg/dL were considered in the normal range according to our institutional laboratory reference values. The following normal range for platelet indices were used as a reference: PLT 150–400,000/mm^3^, MPV 8.6–15.5 fL, plachetocrit-PCT 0.22–0.24%, PDW 8.3–25 fL, and P-LCR 15–35% [10].

Urine complete examination and urine cultures were performed before starting the antibiotherapy for all the patients. We employed urinary tract ultrasound in addition to clinical and biological evaluation of UTI patients (urine cultures and/or suggestive symptoms of UTI). Urine collection was harvested mainly by non-invasive methods, (sterile bags for infants and toddlers and sterile recipients for preschoolers).

The Kass criteria were used to analyze the urine culture: (i) significant bacteriuria/positive urine culture in the context of more than 100,000 colonies/mL of urine; (ii) the absence of UTI in the context of less than 10,000 colonies/mL of urine. For the range between 10,000 and 100,000 colonies/mL of urine we repeated the examination and/or analyzed the case as possible positive diagnosis related to different clinical contexts: (i) false-negative UTI in case of urine collection after the initiation of antibiotic treatment; (ii) the presence of suggestive clinical symptoms (fever, dysuria, pollakiuria, low back pain, etc.); (iii) intermittent acute bacteriuria; (iv) the presence of a microorganism difficult to grow on normal media; (v) the possibility of bacterial inactivation when collecting the sample (use of antiseptic solutions for local toilet before urine culture), diluted urine, abundant rapid flow of urine); (vi) during a recurrence of UTI isolation of a bacterial agent identical to the original one [11,12,13,14]. In a few cases, we performed bladder sampling (catheterization). The reference range for positive UTIs was the presence of more than 10,000 CFU/mL of urine, probe contamination between 10,000 and 1000 CFU/mL and negative urine culture below 1000 CFU/mL [15,16].

### 2.3. Data Analysis

According to our study design, the data on the patient’s demographical and clinical parameters, seizures’ pattern, infectious etiology, biological parameters were analyzed as possible predictors for the UTI status vs. other infections in FS children.

We considered in our analysis the demographical and clinical parameters such as age, gender, FS types (simple or complex), the temperature at seizure onset, time interval from the fever onset to seizure event, clinical symptoms. In addition, we evaluated all the laboratory parameters from venous blood samples: CRP, neutrophils, lymphocytes count, NLR, PCT, P-LCR, PDW, MPV, PLT, red blood cells (RBC), hemoglobin (Hb), hematocrit (Ht), mean corpuscular volume (MCV), mean corpuscular hemoglobin (MCH), the mean corpuscular hemoglobin concentration (MCHC), red cell volume distribution width—coefficient of variation (RDW-CV), red cell volume distribution width—standard deviation (RDW-SD), sodium (Na^+^), chloride (Cl^−^), potassium (K^+^), the logarithmic value of the hydrogen ions concentration (pH), partial pressure of carbon dioxide (pCO_2_), bicarbonate (HCO_3_^−^), and lactate. Further, we compared the previous parameters: (1) in the UTI vs. the non-UTI patients; (2) related to the UTI location; (3) in the upper (U-UTI) vs. lower (L-UTI) urinary tract infection subgroups. The non-UTI patients were subgrouped in acute upper respiratory tract infections (A-RTIs), lower respiratory tract infections patients (L-RTIs), gastroenteritic infections, eruptive infectious diseases, tooth eruption, and omphalitis cases.

Finally, we used the two-step clustering procedure in the exploratory mode to identify subgroups of patients with FS based on the most commonly used inflammatory biomarkers. Among the advantages of this clustering method, we mention: (i) the efficiency in large data sets classification (hierarchical and k-means clustering methods do not scale efficiently in large data sets); (ii) ability to create groups using variables of mixed data types (categorical and continuous); (iii) automatically detection/selection of the number of clusters/subgroups.

The two-step cluster algorithm is a hybrid approach between the K-means cluster method and hierarchical cluster method [8,17]. This algorithm uses (i) a distance measure of similarity/dissimilarity to separate individuals (log-likelihood distance if one or more variables are categorical, Euclidean distance if all of the variables are continuous), and (ii) a method to choose the optimal number of clusters (BIC—Schwarz’s Bayesian Criterion or AIC—Akaike Information Criterion).

We computed the mean, standard deviation (SD), median, and interquartile range (IQR) values for the continuous variables while the categorical variables were summarized using counts and percentages. Shapiro–Wilk test and histograms were used to examine the data normality. Chi-square analysis or Fischer exact test, Mann–Whitney, and Kruskal-Wallis tests were applied (depending on variables type and distribution). The goal was to assess group and subgroup comparisons (UTI vs. non-UTI, U-UTI vs. L-UTI, between the emerged clusters). The cut-off values of CRP for the non-UTI and UTI groups’ differentiation were computed using ROC analysis and Youden index (area under the curve—AUC and 95% confidence intervals—CI). Analysis was conducted using SPSS v.20 (SPSS Inc., Chicago, IL, USA) and R software. Statistical difference was considered for *p* < 0.05.

## 3. Results

### 3.1. General Description

We enrolled in the studied group 136 children, with an average age of 23.23 ± 12.43 months and a balanced gender distribution (50.8% boys). There were 197 different FS events, 156 (79.2%) with simple FS and 41 (20.8%) with complex patterns. The main seizure duration ranged between 1–5 min. From the total FS events, 58.3% were preceded by temperatures higher than 39 °C. Only in a small number of cases (4.1%) we noticed a higher than 72 h time interval from fever occurrence to seizure onset.

The acute upper respiratory tract infections (A-RTIs) were identified as the most common cause of fever in our study group (72.1%), followed by acute lower respiratory tract infections (L-RTIs, 14.2%) and UTIs (10.2%). Other fever precipitating factors in the non-UTI group were due to gastroenteritic infections (2%), eruptive infectious diseases (0.5%), tooth eruption (0.5%), and omphalitis (0.5%). The UTIs (20 cases) represented 14.4% of the total bacterial infection cases (139 cases). In addition, UTIs succeeded A-RTIs as the second most common bacterial etiology of FS (71.9%). There were only four patients with a history of recurrent UTIs. We documented only one case of bilateral (congenital) hydronephrosis on the ultrasound screening for urinary tract malformations.

Complex febrile seizures were reported in a higher percentage in UTI children (35%) compared to the non-UTI group (gastroenteritis subgroup 25%, L-RTI subgroup 21.43%, A-RTI subgroup 19.01%).

### 3.2. Clinical Data Comparison: UTI versus Non-UTI Groups and U-UTI versus L-UTI Subgroups

We recorded 20 UTI (10.2%) cases (mainly in boys) and 177 (89.8%) cases in the non-UTI groups (mostly in girls). The groups were age-balanced (24.00 ± 16.09 months vs. 23.14 ± 12.00 months). The age range between 13–24 months was associated with the peak incidence of both UTI and non-UTI pathology.

There was a homogeneous distribution of cases related to the (low-grade, moderate or high) fever degree in the UTI group at the time of the seizure event. Patients included in the non-UTI group (61%) exhibited high fever or hyperpyrexia >39 °C and a higher average temperature compared to UTI patients (35%). In both groups, seizures events were preceded mainly by a short time interval of fever. Only a small number of FS events, 15% in the UTI group and 11.4% in the non-UTI group, were preceded by a fever interval longer than 24 h. In more than one-third of the UTI children, we noticed a nonspecific clinical picture confined to fever and FS. Urinary symptoms were reported in a small number of UTI cases (20%).

Complex febrile seizures and febrile seizures recurrence within the first 24 h were reported higher in children from the UTI group (35%) vs. the non-UTI group (19.21%). A detailed analysis for the demographics and clinical symptoms is presented in Appendix B Table A2.

In UTI subgroups, there was a higher prevalence of U-UTIs with 13 cases (54.2%), mainly in girls. For U-UTIs, the FS age range was similar to the one recorded considering the whole cohort. The recorded peak incidence was noticed in the age range of 13–24 months (53.8%). Complex febrile seizures were reported in a higher percentage in the L-UTI subgroup compared to the U-UTI subgroup (42.86% vs. 30%). Only one patient from the U-UTI subgroup and another patient from the L-UTI subgroup associated prolonged febrile seizures (longer than 15 min). Then, a significant number of patients with U-UTIs were oligosymptomatic, presenting nonspecific symptoms limited to fever and FS compared to the L-UTI subgroup (46.2% vs. 14.3%). To conclude, an important number of UTI children presented complex febrile seizures, with an initial elusive clinical picture in a considerable number of patients with U-UTIs.

### 3.3. Laboratory Parameters Comparison in UTI versus Non-UTI Groups and U-UTI versus L-UTI Subgroups

In our cohort, the CRP values were higher in the UTI group compared to the non-UTI group (30, IQR 10–46 vs. 15, IQR 5–35 mg/dL, *p* = 0.054) (Appendix B, Table A2). Furthermore, the CRP values were significantly higher in children with U-UTIs compared to the non-UTI subgroups (30, IQR 13–46 vs. 14.50, IQR 6–34 mg/dL, *p* = 0.028). Further CRP analysis set out a cut-off point of 25.8 g/L (AUC = 0.681, 95% CI (0.539–0.823), *p* = 0.030) to differentiate the U-UTI cases from other subgroups. In UTI cases, a cut-off point of 21.5 g/L for CRP set apart the U-UTI patients from the L-UTI patients (AUC = 0.654, 95% CI (0.385–0.922), *p* = 0.293).

Neutrophils count were higher in the U-UTI patients than in the L-UTI patients (10.72, IQR 7.54–14.31 vs. 6.98, IQR 4.22–8.57, *p* = 0.054), while NRL values were similar in both UTI subgroups (4.26, IQR 2.38–5.37 vs. 3.64, IQR 2.77–8.12, *p* = 0.930). The cases presenting acute reactive thrombocytosis were noticed only in the U-UTI subgroup. The assessment of erythrocyte indices values, platelet indices, and hydroelectrolytic or acid-base status did not point out statistically significant differences between groups and the subgroups of patients (UTI vs. non-UTI and U-UTI vs. L-UTI, respectively).

Regardless of the underlying infectious context the mean values of plasma Na^+^ were suggestive for mild hyponatremia, while the mean pH, pCO_2,_ and HCO_3_^−^ values suggested alkalosis. In the UTI group, all patients presented exclusively respiratory alkalosis. In the non-UTI group, minimum pH values of 7.21, minimum pCO_2_ of 13.90 mm/Hg, or minimum HCO_3_ of 24.90 mmol/L were suggestive for metabolic acidosis and were reported in patients with gastroenteritis.

The most frequently isolated pathogens in the urine cultures were Gram-negative bacilli (80%) with *Escherichia coli* documented in 50% of cases (10 cases), Proteus mirabilis 25% (4 cases), and Enterobacter spp. 10% (2 cases). Gram-positive cocci (*Enterococcus* species) were isolated in 4 cases. From these 20 UTI cases, *Escherichia coli* represented the prevalent pathogenic bacteria in the U-UTIs (69.2%) and Proteus mirabilis in L-UTIs (57.1%). Enterobacter cloacae was rarely isolated regardless of the site of infection (7.7% in U-UTIs vs. 14.3% in L-UTIs). The urine cultures revealed a bacterial load of more than 10^5^ CFU/mL of urine in 75% (15 cases): (i) all 10 cases with *Escherichia coli* (50%); (ii) 1 case with Proteus mirabilis (5%); (iii) 2 cases with *Enterococcus* spp. (10%); and (iv) 2 cases with Enterobacter cloacae (10%). The rest of 25% (5 cases) had a bacterial load of more than 10^4^ CFU/mL of urine in a clinical suggestive context and presented the following distribution: (i) 3 cases with Proteus mirabilis (15%); (ii) 2 cases with *Enterococcus* spp. (10%).

To analyze the cases’ distribution based on pathogen strains and urine leukocytes/nitrites stick test, we considered the 2 years of age as a cut-off point according to the data presented by Coulthard in a recent metanalysis (2019) on this topic [6]. The pathogens distribution based on the aforementioned cut-off point revealed that 70% of the cases were aged <2 years, and only 30% of the cases aged >2 years. For the first subgroup: (i) 8 cases (20%) presented with *Escherichia coli* (1:1 female: male gender ratio); (ii) 2 male cases (10%) with Proteus mirabilis; (iii) 2 male cases (10%) with *Enterococcus* spp.; (iv) 2 cases with Enterobacter cloacae (1:1 female:male gender ratio). For the second subgroup: (i) 2 female cases (10%) with *Escherichia coli*; (ii) 2 cases (10%) with *Proteus mirabilis* (1:1 female: male gender ratio); and (iv) 2 cases with *Enterococcus* spp. (1:1 female: male gender ratio).

The urine nitrite stick test was positive only in 4 cases (20%), and all of them had *Escherichia coli* as bacterial load with more than 10^5^ CFU/mL of urine (2 female gender cases aged <2 years and 2 male gender cases aged >2 years). Positive urine leukocyte stick test results were recorded in 9 cases (45%), and all of them had positive urine cultures with a bacterial load of more than 10^5^ CFU/mL of urine: (i) 4 cases with *Escherichia coli* (3 cases aged <2 years, 2:1 female: male ratio, 1 male case aged >2 years); (ii) 1 male case with Proteus mirabilis aged <2 years; (iii) 2 male cases with *Enterococcus* spp. and equally distributed to the age intervals; (iv) 2 cases with Enterobacter cloacae aged <2 years and 1:1 female: male gender ratio.

### 3.4. Two Steps Cluster Analysis Results

**Figure 1:**
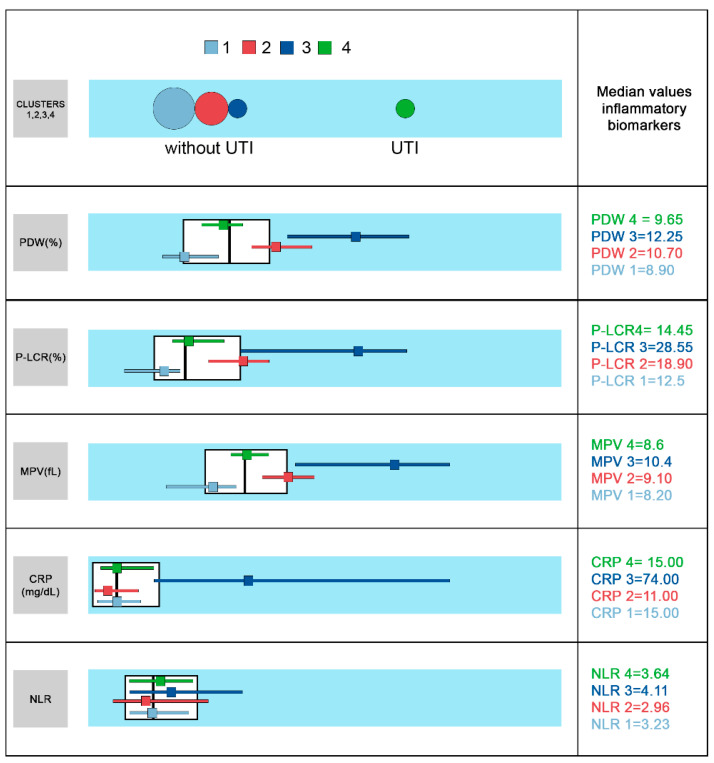
Two step cluster analysis for the whole cohort of patients, using as segmentation variables the proposed inflammatory biomarkers. Clusters 1, 2, 3, and 4 are color-coded and the median values for PDW (%), P-LCR (%), MPV (fL), CRP (mg/dL), and NLR are accordingly presented for each cluster.

In this study we performed a two-step cluster analysis for the whole cohort of patients, using as segmentation variables the inflammatory biomarkers CRP, NLR, P-LCR, MPV, PDW, and PLT. The algorithm generated four distinct groups of patients (see Figure 1). The differences between clusters were based on CRP, P-LCR, MPV, PDW, PLT (*p* = 0.042), and less on NLR (*p* = 0.462).

The UTI group (cluster 4) showed the highest PLT values (331, IQR 261-495) and intermediate CRP (15, IQR 7–32), PDW (9.65, IQR 9.20–10.10), P-LCR (14.45, IQR 12.80–17.70), MPV (8.60, IQR 8.40–8.90) and NRL values (3.64, IQR 2.03–5.37). Cluster 3, gathered only the patients with respiratory bacterial infections (lower and upper). This cluster’s profile unveiled the highest CRP (74, IQR 26–171 g/L), PDW (12.25, IQR 10.70–13.40), MPV (10.40, IQR 8.90–11.20), P-LCR (28.55, IQR 16.30–32.80), NLR (4.11, IQR 2.11–7.58) and lowest PLT values (218.50, IQR 170–304).

Several parameters set apart these 2 clusters, both with bacterial etiology: CRP (*p* = 0.021), PDW, P-LCR (*p* = 0.001), MPV (*p* = 0.000) and PLT (*p* = 0.033).

Clusters 1 and 2 were represented mainly by upper respiratory infections and other infections (viral or bacterial). Cluster 1 was the largest one, including almost 50% of all patients with the lowest PDW (8.90, IQR 8.50–9.60), MPV (8.20, IQR 7.60–8.50), P-LCR (12.50, IQR 9.70–13.80) values. A detailed description of the four clusters is presented in Table 1.

The demographic and clinical cluster profile revealed male predominance in cluster 4 (UTI) while female gender prevailed in cluster 3 (respiratory bacterial) (Appendix B, Table A1). The maximum age group incidence was 13–24 months in both clusters. Hyperpyrexia was noticed in a higher percentage in cluster 3 patients (respiratory bacterial infections) than in cluster 4. Most of the patients in cluster 4 had a moderate febrile rise at seizure onset (between 38–39 °C). The percentage was slightly lower in patients with respiratory bacterial infections (cluster 3). In both clusters, we noticed similar trends: (1) most of the seizure events lasted between 1 and 5 min; (2) 21.43% and 14.29% of the patients had prolonged seizures (duration > 15 min); (3) the main febrile seizures type was simple FS (64.29% from the whole cohort); (4) there was a similar percentage of recurrence of seizures within the first 24 h.

## 4. Discussion

We report a UTI prevalence of 10.7%, comparable to Abedi et al.’s (11.1%) and Kazeminezhad et al.’s (11.28%) research [18,19]. Shaikh et al.’s meta-analysis on pediatric UTIs highlights a general prevalence of UTIs of 7.0% (CI: 5.5–8.4) [20]. There is a low frequency of febrile seizures and UTIs in infants, only five cases. We could not confirm in our study the UTI bimodal age model proposed in Leung et al.’s meta-analysis. This model refers to a peak in the first year of life and another peak between 2–4 years, corresponding to the age of toilet training [18,19,20,21,22,23,24].

During the first year of life, in uncircumcised infants aged under 6 months, there is a higher incidence of UTIs, according to Wiswell et al.’s data, with a 10–12 times higher risk of UTIs [25]. Shaikh et al.’s meta-analysis showed an age-related decline in the prevalence of UTI in males [20].

Regarding UTI subgroup prevalence, our study revealed 65% of U-UTIs similar to that reported by Mahyar and colleagues on a group of 25 UTI children with FS (68%) [26]. U-UTIs are prevalent at a younger age, representing 69.23% of UTIs cases under two years of age.

When it comes to etiology, our data correlated with the results of other epidemiological studies. We confirm the most common etiological UTIs pathogens of enteric origin [27,28,29]. The most frequently isolated pathogens in the urine culture were *Gram-negative bacilli* (80%), *Escherichia coli* (45%), *Proteus mirabilis* (25%), and *Enterobacter* spp. (10%). We are in agreement with Srinivas et al.’s reports. These authors mentioned 50% for *E. coli* infection and 11.11% for Proteus and Citrobacter in children with UTIs and FS. Bryan C.S et al. reported *E. coli* as a pathogen in 85% of cases, while according to Bagga et al., 90% of the symptomatic UTIs and 70% of the recurrent UTIs were due to the same pathogen [28,29,30,31,32]. Then, based on UTI classification, we also report *E. coli* as the most common bacterial agent in U-UTIs (69.2% of patients). We are in line with other studies presenting *E. coli* pathogen to be responsible for 80–90% of acute episodes of acute pyelonephritis in the community, especially in children. Less common pathogenic bacteria included *Proteus mirabilis*, *Klebsiella* spp., and *Staphylococcus saprophyticus* [32,33,34,35]. The etiology of the L-UTIs was dominated by Proteus mirabilis (57.1%), a pathogen more commonly associated with UTIs in boys than in girls [36,37,38]. Furthermore, our findings related to risk factors for the FS events were similar to other authors’ reports [8,9]. In the UTI group, all the patients presented respiratory alkalosis, while in the non-UTI group, the acid-base status workup showed metabolic acidosis in patients with gastroenteritis [8,9].

In a clinical context of nonspecific symptoms, especially at an early age, a rapid diagnosis of UTI may be difficult. In our study, one-third of the patients with UTI and febrile seizures were oligo symptomatic, presenting only with fever, while urinary symptoms were reported in 20% of the patients. Our study results correlate with the literature, which mentions unexplained fever as the most common, sometimes the only symptom of UTI in the first two years of life [35,38,39,40,41].

High CRP values are common findings in UTI; however, CRP is a nonspecific marker of inflammation related to the etiology [42,43]. CRP is an acute-phase reactant, a sensitive indicator of inflammation and one of the most commonly used biomarkers in current medical practice. Its rapid production in the liver following a tissue lesion reaches a maximum level in 48 h and then decreases rapidly [42]. In our study, the CRP values and neutrophil count in children with U-UTIs were significantly higher than those with other triggers for fever or L-UTIs. Based on our findings, we propose a cut-off point of 25.8 g/L for CRP to differentiate the U-UTI group from other causes of fever, while a cut-off point of 21.5 g/L for CRP differentiates the U-UTI from the L-UTI patients. Similarly, CRP cut-off points of 20 mg/DL–20.5 mg/dL were reported by Fretzayas et al., Leroy et al., and Pecille et al., while Ansar-Gilani et al. reported a cut-off value of 30 mg/L between U-UTIs and L-UTIs. Nevertheless, a wide range of cut-off points, varying between 1 and 88 mg/dL, were described in Zhang et al.’s meta-analysis [44,45,46,47,48]. Moreover, according to Shaikh et al.’s opinion, the CRP’s sensitivity (cut-off value 20 mg/L) to predict U-UTIs is high (94%), but specificity varies (39%) [49,50]. We have to mention, though, that the number of studies in this systematic review was limited and the heterogeneity substantial. The specificity and positive predictive values varied because CRP values were also increased in children with L-UTIs. This suggests a possible role for other inflammatory markers in distinguishing UTIs (upper and lower UTI), and the literature is scarce in this respect [49,50]. Furthermore, these remarks could explain a wide range of cut-off points, varying between 1 and 88 mg/dL, reported in the meta-analysis of Zhang et al. [45,46,47,48]. On the same note, we interpreted the tendency towards statistical significance (*p* = 0.054) for a higher neutrophils count in the U-UTI patients compared with the L-UTI patients (Table A2). This usually correlates with more severe clinical symptoms in the U-UTIs vs. L-UTIs. Our results are in line with Lee J.W et al. reports 2016 [51] and might offer a clue also for olygosimptomatic FS patients (U-UTIs vs. L-UTIs). Lee et al. presented higher values for CRP, WBC, and the fraction of circulating immature granulocytes in acute pyelonephritis than in lower UTI (*p* < 0.01), as a consequence of the bacterial infections’ severity. The urine dipstick tests results for the UTI cases confirmed the recent reports presented in Coulthard’s metanalysis, stating that “urine nitrite stick tests miss UTIs in about three-quarters of children aged <2 years probably due to a combination of low dietary nitrate, the instability of their urinary nitrite, and urinary frequency” [6]. In our UTI cases, only 4 out 14 patients aged <2 years presented positive nitrite tests. The presence of urine leukocytes was noticed only in children with a bacterial load of more than 10^5^ CFU/mL of urine. Nevertheless, from the total of 14 patients in this category, only 9 patients had leukocytes in the urine. Further analysis on larger datasets to correlate leukocytes’ presence with significant bacteriuria and other urine biomarkers in olygosimptomatic FS patients is needed to explore a possible pattern in this respect.

A significant number of UTI children presented complex febrile seizures, with an initial elusive clinical picture in a considerable number of patients with U-UTIs. In this context, new approaches related to clusters of biomarkers could improve the timing of the etiological diagnosis, treatment, and the early prognosis of febrile seizures associated with UTI. While the analysis of individual inflammatory parameters provided limited knowledge on differentiating UTIs from other localized infections, the cluster analysis did identify four clusters with distinct inflammatory patterns in relation to the etiology of the infectious context. Severely raised CRP values were noticed in cluster 3 with bacterial respiratory infections. Compared to the other clusters, the inflammatory pattern revealed the highest CRP, PDW, MPV, PLT, P-LCR, and NRL values and the lowest PLT values. The UTI cluster (4) showed slightly raised CRP values (up to two times the normal range), normal PDW values, median values of P-LCR and MPV at the lower limit of the normal range, and mean PLT values at the upper limit of the normal range. Our findings suggest that the UTIs seem to be highly unlikely in patients with bacterial infections and with a specific inflammatory pattern consisting of severely raised CRP values and platelet indices within the normal range.

At a glance, the two step cluster analysis unsupervised machine learning approach could offer a practical model to hasten the etiology diagnosis in children with febrile seizures. However, there are some limitations to our proposed model. First, it was a prospective study with its limitations in terms of selection bias compared to randomized control trials. Then, there were only 20 patients with UTIs. The sample size limited further analysis for a possible statistical significance regarding the correlation between bacterial load (CFU/mL) with the occurrence and severity of FS. The bacterial respiratory infections were mainly grouped in cluster 3 and had a distinctive inflammatory pattern. Nevertheless, some of the cases were cast in clusters 1 and 2 along with other infection types (omphalitis, gastroenteritis) irrespective of the etiology (viral vs. bacterial). We believe that these limitations recommend future multicentric randomized-controlled trials conducted on more extensive data samples that should be employed to improve this model’s accuracy and provide specific profiles for other infection sites. Combined approaches (different cytokines clusters) correlated with specific cut-off points for CRP, PDW, MPV, PLT, P-LCR, MPV and NLR on big datasets or clustered with these nonspecific biomarkers might offer a solution in this respect [5]. Moreover, future research approaches might also address the correlations between the amount of CFU/mL and the occurrence and severity of FS.

## 5. Conclusions

Although the analysis of individual inflammatory parameters provided limited data in relation to the etiology of the febrile context, using two step cluster analysis offered a distinct inflammatory pattern has emerged. This pattern is defined by higher PDW, P-LCR, MPV, CRP, and NLR, and lower PLT in UTIs and A-RTIs. Furthermore, these patients with suggestive bacterial context, characterized by severely raised CRP values and platelet indices within normal range, were associated mainly with bacterial respiratory infections. In addition, for these patients, UTIs were highly unlikely. Therefore, considering the nonspecific clinical picture of UTIs in childhood, the combined analysis of inflammatory parameters targeted on patients with bacterial infections could provide a clue in selecting the patients with s probable UTI.

## Figures and Tables

**Table 1:** Description of the four clusters identified by the two step cluster algorithm.

	Cluster 1	Cluster 2	Cluster 3	Cluster 4	*p*
**CRP**	22.87 ± 26.24	18.13 ± 0.02	92.36 ± 0.35	21.29 ± 0.90	0.000
	(2.00–114.00)	(4.00–75.00)	(4.00–209.00)	(4.00–52.00)	
	15.00	11.00	74.00	15.00	
	(5.00–26.00)	(5.00–26.00)	(26.00–171.00)	(7.00–32.00)	
**PDW**	8.97 ± 0.70	10.91 ± 2.97	12.31 ± 1.01	9.51 ± 8.79	0.000
	(7.00–10.20)	(9.20–13.20)	(9.10–16.30)	(7.80–10.70)	
	8.90	10.70	12.25	9.65	
	(8.50–9.60)	(10.20–11.40)	(10.70–13.40)	(9.20–10.10)	
**P-LCR**	11.85 ± 2.91	19.13 ± 8.70	27.21 ± 9.26	14.79 ± 6.85	0.000
	(6.20–20.50)	(11.80–27.20)	(13.90–44.10)	(6.60–21.50)	
	12.50	18.90	28.55	14.45	
	(9.70–13.80)	(15.90–20.80)	(16.30–32.80)	(12.80–17.70)	
**MPV**	8.06 ± 0.54	9.14 ± 0.52	10.24 ± 1.30	8.59 ± 0.68	0.000
	(6.70–9.20)	(8.20–10.40)	(8.10–12.40)	(7.10–9.70)	
	8.20	9.10	10.40	8.60	
	(7.60–8.50)	(8.80–9.40)	(8.90–11.20)	(8.40–8.90)	
**PLT**	345.06 ± 0.75	326.95 ± 0.78	271.36 ± 0.00	395.29 ± 0.77	0.042
	(111.00–650.00)	(106.00–665.00)	(120.00–655.00)	(210.00–716.00)	
	322.00	284.00	218.50	331.00	
	(277.00–411.00)	(247.00–393.00)	(170.00–304.00)	(261.00–495.00)	
**NLR**	4.18 ± 5.69	4.03 ± 2.33	5.77 ± 6.75	4.07 ± 1.80	0.462
	(0.56–22.75)	(0.27–13.64)	(0.60–22.35)	(0.14–10.71)	
	3.23	2.96	4.11	3.64	
	(1.79–5.03)	(1.44–6.47)	(2.11–7.58)	(2.03–5.37)	

Data are presented as mean ± standard deviation (minim–maxim), median (IQR—interquartile range).

**Figure A1:**
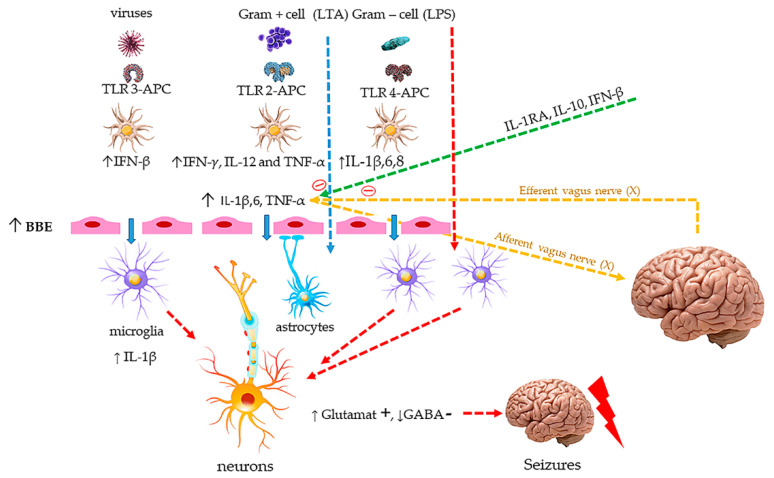
Pathogenic mechanisms of FS in different infections (viral and bacterial-Gram positive and Gram-negative).

## Data Availability

The data presented in this study are available on request from the corresponding author. The data are not publicly available due to data protection legislation.

## Appendix A

Viral and bacterial infections can trigger FS events in genetically susceptible children [4,52]. Genetic variants as single nucleotide polymorphism (SNPs) have been described both for pro-inflammatory and anti-inflammatory cytokines. It seems that high levels of pro-inflammatory cytokines, unbalanced by their anti-inflammatory pairs in the blood, enhance blood-brain barrier (BBE) permeability with microglia activation (Figure A1). In turn, activated microglia release particularly IL-1β, which further triggers prostaglandin 2 (PGE2) synthesis and consequently a rapidly rising fever. Moreover, elevated levels of IL-1β increase glutamatergic neurotransmitters (excitatory) and abate GABA-ergic (inhibitory) neurotransmission [4,52,53]. Eventually, the seizure event ensues [52].

The most referred pro-inflammatory cytokines in the literature are IL-1β, IL-6, IL-8, IL-12, IL-22, (TNF)-α, IFN-γ, (TGF)-β, high mobility group box 1 protein (HGMB1) while the anti-inflammatory cytokines are IL-1RA, IL-10, and IFN-β. IL-1β pairs with IL-1RA, TNF-α and IFN-γ with IL-10. IFN-β reduces BBE permeability to cytokines and inflammatory cells and the neuroinflammation [54]. In addition, cholinergic neurotransmission signals in the efferent vagus nerve, along with anti-inflammatory cytokines, regulate the inflammation with negative feedback. This control seems to be lost in genetic susceptible children with FS [4,52,53].

Current research refers to different innate immune responses related to viral infections versus bacterial infections. Moreover, there is a different pattern of inflammation response to Gram-negative pathogens compared to Gram-positives due to molecular fingerprints on pathogens’ structure such as lipopolysaccharide (LPS) on Gram-negative cell-wall and lipoteichoic acid (LTA) in Gram-positive bacteria (Figure A1). LPS and LTA increase BBE permeability, especially LPS. In viral infections, there is a Toll-like receptor-3 (TLR3) activation on monocytes and macrophages suggesting a particular host response to viruses, and IFN-β is a marker of (TLR3) [51]. In bacterial infections, LTA is recognized by (TLR2) and LPS by (TLR4) (Figure A1) [5]. In children with UTIs, the etiology is dominated by Gram-negative bacteria. In cases with asymptomatic bacteriuria, TLR4 genes mutations were detected, hence, a specific impairment in innate immune response could influence the host susceptibility to the infection type [29,35]. Furthermore, in vivo and in vitro studies revealed differences in cytokine responses in Gram-positive and Gram-negative bacteria. Higher levels of IFN-γ, IL-12 and TNF-α were noticed in infections with Gram-positive bacteria while Gram-negative bacteria triggered higher levels of IL-6, IL-8, and IL-10 [5,55].

Clinical studies on children with FS showed promise in validating specific patterns of pro-inflammatory cytokines correlated with the type of infection, yet these biomarkers are expensive. Nevertheless, current research trends highlight the importance of specific indices such as neutrophil-to-lymphocyte ratio (NLR), platelet count (PLT) ratio, mean platelet volume (MPV) and platelet count, and red blood cell distribution width (RDW). These low-cost inflammatory biomarkers could offer interesting perspectives in FS research to forecast specific outcomes. According to Liu et al., 2018, NLR and MPV might predict the FS occurrence [3].

Recently, Rini et al., 2020, reported in a prospective study in adults, higher values of MLR (monocytes-to-lymphocytes ratio) and NLR in bacterial infection cases and lower values for platelet–lymphocyte ratio (PLR) in viral infections [56]. Novel research approaches, according to Heffernan et al., 2021, might address both new and classical biomarkers as independent variables (predictors) in machine learning algorithms to forecast the infection type or the site location for the infection [57].

Potential therapies to address seizures cases in childhood that end in refractory epilepsy envisage nervous vagus stimulation (VNS) to reduce the pro-inflammatory cytokines and neurofeedback protocols to modulate the seizures activity. Current guidelines in VNS, which is an invasive procedure, suggest its usefulness in pediatric patients with intractable epilepsy that are resistant to antiepileptic drugs [58]. VMS works on stimulation frequency on the left vagus nerve with a specific pulse generator device set to a 20–30 Hz pulse frequency with a pulse width of 250–500 µs [59]. These patients represent around one-third of the epileptic children, and it seems that VNS holds promise in reducing more than half of the seizure events frequency in almost 50% of the studied patients after 2 years of follow-up [58]. The neurofeedback approach, however, is a non-invasive approach based on operant conditioning [60]. While the EEG signal is recorded, its particular metrics to be trained (power, amplitude, frequency) are analyzed by the computer providing feedback in real-time to the subject [60,61]. As the signal is modulated toward the desired goal by comparison to the normative database embedded in the software, some “rewards” consisting of visual and/or auditory stimuli are offered to the trained subject. Different neurofeedback protocols to modulate EEG wave bands (delta; theta; alpha; beta) have been proposed. The sensorimotor rhythm (SMR), 12–15 Hz, associated with the sensorimotor cortex is correlated with alertness and states of immobility. In epileptic patients SMR is decreased while theta rhythm (4–9 Hz) is increased. The goal in these patients is SMR stimulation and theta wave activity inhibition. Currently both VNS and neurofeedback are considered encouraging complementary non-pharmacological interventions for intractable epilepsy, which might alleviate the seizure events frequency for this pediatric population [62,63].

## Appendix B

**Table A1:** Description of the demographic and clinical data.

		Non-UTI 177 (89.8%)	UTI 20 (10.2%)	*p*	U-UTI 13 (65%)	L-UTI 7 (35%)	*p*
Gender	M	88(49.72)	12(60.00)	0.383	6(46.15)	6(85.71)	0.085
	F	89(50.28)	8(40.00)		7(53.85)	1(14.29)	
Age	<6	5(2.82)	0(0.00)	0.294	0(0.00)	0(0.00)	0.061
	6–12	24(13.56)	5(25.00)		2(15.38)	3(42.86)	
	13–24	81(45.76)	8(40.00)		7(53.85)	1(14.29)	
	25–36	41(23.16)	2(10.00)		0(0.00)	2(28.57)	
	>36	26(14.69)	5(25.00)		4(30.77)	1(14.29)	
	M ± SD	23.14 ± 12.01	24 ± 16.10		25.77 ± 17.89	20.71 ± 12.70	
Temperature	<38	20(11.30)	6(30.00)	0.106	4(30.77)	2(28.57)	0.737
	38–39	49(27.68)	7(35.00)		5(38.46)	2(28.57)	
	39–40	78(44.07)	6(30.00)		3(23.08)	3(42.86)	
	40–41	25(14.12)	1(5.00)		1(7.69)	0(0.00)	
	>41	5(2.82)	0(0.00)		0(0.00)	0(0.00)	
FS episode Type	S	143(80.79)	13(65.00)	0.099	9(69.23)	4(57.14)	0.589
	C	34(19.21)	7(35.00)		4(30.77)	3(42.86)	
Seizure duration	<1	25(14.12)	4(20.00)	0.843	2(15.38)	2(28.57)	0.731
	1–4.9	84(47.46)	9(45.00)		7(53.85)	2(28.57)	
	5–14.9	55(31.07)	5(25.00)		3(23.08)	2(28.57)	
	≥15	13(7.34)	2(10.00)		1(7.69)	1(14.29)	
Recurrence/24 h	Yes	15(8.47)	3(15.00)	0.337	2(15.38)	1(14.29)	0.948

Seizure duration was measured in minutes. The *p*-value was computed using Chi-square analysis, Mann–Whitney or Kruskal–Wallis tests (as appropriate, depending on variables type and distribution).

**Table A2:** Description of the laboratory data.

	Non-UTI	UTI	*p*	U-UTI	L-UTI	*p*
CRP	27.21 ± 35.85	38.05 ± 42.30	0.054	45.92 ± 48.26 (4–171) 30 (13–46)	21.00 ± 18.48	0.292
	(2–209)	(4–171)			(5–52)	
	15	30			14.50	
	(5–35)	(10–46)			(6–34)	
PDW	9.95 ± 1.54	9.78 ± 1.00	0.941	9.85 ± 1.04 (8.20–12.20) 9.70 (9.20–10.20)	9.65 ± 1.01	0.960
	(7–16.30)	(7.80–12.20)			(7.80–10.70)	
	9.80	9.70			9.85	
	(8.80–10.60)	(9.40–10.20)			(9.50–10.20)	
P-LCR	15.82 ± 6.43	16.02 ± 5.25	0.415	16.47 ± 5.55	15.13 ± 4.97	0.963
	(6.20–44.10)	(6.60–31.90)		(10.20–31.90)	(6.60–21.50)	
	14.20	15.65		15.65	15.45	
	(11.90–19.00)	(14.10–17.70)		(13.50–17.65)	(14.10–17.70)	
MPV	8.63 ± 0.95	8.73 ± 0.82	0.442	8.78 ± 0.82	8.63 ± 0.88	0.880
	(6.70–12.40)	(7.10–10.80)		(7.90–10.80)	(7.10–9.70)	
	8.50	8.60		8.60	8.70	
	(8.10–9.10)	(8.40–8.90)		(8.40–8.90)	(8.40–9.20)	
PLT	327.41 ± 118.03	355.75 ± 163.13	0.711	374.62 ± 188.19	320.71 ± 105.99	0.606
	(98–665)	(120–716)		(120–716)	(188–461)	
	303	315.50		326	305	
	(247–391)	(228.50–478)		(237–518)	(210–450)	
Neutrophils	8.88 ± 5.87	9.57 ± 4.92	0.341	10.87 ± 5.25	6.75 ± 2.62	0.054
	(0.88–38.16)	(0.23–21.17)		(0.23–21.17)	(3.44–10.31)	
	7.83	8.59		10.72	6.98	
	(4.73–11.38)	(6.61–13.86)		(7.54–14.31)	(4.22–8.57)	
Lymphocytes	2.98 ± 1.94	2.59 ± 1.67	0.328	3.02 ± 1.83	1.67 ± 0.75	0.136
	(0.54–13.74)	(0.62–6.43)		(0.62–6.43)	(0.80–2.73)	
	2.52	2.27		2.77	1.58	
	(1.75–3.64)	(1.27–4.11)		(1.55–4.31)	(1.07–2.27)	
NLR	3.93 ± 3.33	5.36 ± 5.10	0.171	5.49 ± 5.81	5.07 ± 3.56	0.930
	(0.21–22.75)	(0.14–22.35)		(0.14–22.35)	(1.55–10.71)	
	3.04	4.06		4.26	3.64	
	(1.59–5.08)	(2.38–5.89)		(2.38–5.37)	(2.77–8.12)	
RBC	4.46 ± 0.40	4.50 ± 0.36	0.898	4.55 ± 0.34	4.38 ± 0.40	0.254
	(3.27–6.22)	(4.03–5.26)		(4.07–5.26)	(4.03–5.05)	
	4.46	4.42		4.45	4.26	
	(4.23–4.70)	(4.25–4.69)		(4.33–4.69)	(4.05–4.63)	
Hb	11.32 ± 1.31	11.08 ± 1.38	0.487	10.97 ± 1.55	11.32 ± 0.99	0.930
	(5.10–13.90)	(7.80–13.10)		(7.80–13.10)	(9.80–12.90)	
	11.45	11.40		11.40	11.20	
	(10.70–12.10)	(10.40–11.80)		(10.40–11.80)	(11.20–11.60)	
Ht	32.93 ± 2.98	32.12 ± 3.02	0.253	32.16 ± 3.45	32.03 ± 2.07	0.861
	(19.70–40.00)	(26.10–37.90)		(26.10–37.90)	(29.70–35.30)	
	32.95	31.70		32.40	31.70	
	(31.10–34.90)	(30.30–34.60)		(30.60–34.60)	(30.30–33.50)	
MCV	73.52 ± 6.31	71.58 ± 6.37	0.305	70.68 ± 7.15	73.74 ± 3.65	0.598
	(51.90–89.00)	(59.00–78.20)		(59.00–77.20)	(69.10–78.20)	
	73.90	74.10		74.65	72.40	
	(70.90–77.50)	(69.10–76.10)		(63.40–75.80)	(72.40–76.60)	
MCH	25.51 ± 2.94	24.69 ± 2.93	0.244	24.15 ± 3.34	25.88 ± 1.33	0.510
	(13.70–32.40)	(17.60–27.80)		(17.60–27.70)	(24.20–27.80)	
	25.80	25.60		26.10	25.55	
	(24.40–27.25)	(24.20–26.50)		(21.90–26.40)	(25.10–27.10)	
MCHC	34.21 ± 2.17	34.39 ± 1.79	0.891	33.98 ± 1.83	35.28 ± 1.42	0.095
	(23.10–37.30)	(29.90–37.00)		(29.90–36.40)	(33.00–37.00)	
	34.60	34.60		34.30	35.30	
	(33.55–35.40)	(33.00–35.30)		(33.00–34.90)	(34.60–36.50)	
RDW-CV	14.63 ± 2.38	14.72 ± 1.92(12.50–19.60) 14.60(13.20–16.40)	0.716	15.11 ± 1.99	13.88 ± 1.59	0.160
	(8.00–26.90)			(13.00–19.60)	(12.50–16.60)	
	14.10			14.70	13.35	
	(13.20–15.20)			(13.60–16.40)	(12.60–14.90)	
RDW-SD	38.14 ± 4.18	37.18 ± 3.10	0.316	37.61 ± 2.86	36.25 ± 3.66	0.219
	(32.50–73.40)	(33.00–43.10)		(33.20–43.10)	(33.00–42.70)	
	37.60	36.70		37.50	34.95	
	(35.80–39.40)	(34.60–39.70)		(35.00–39.70)	(33.70–38.20)	
Na^+^	130.05 ± 2.82	129.89 ± 3.02	0.744	130.08 ± 3.58	129.57 ± 1.90	0.966
	(122–140)	(126–135)		(126–135)	(126–132)	
	130	129		129	130	
	(128–132)	(128–132)		(127–133.50)	(129–131)	
Cl^−^	107.54 ± 3.40	106.58 ± 4.21	0.19	106.54 ± 3.86	106.67 ± 5.24	0.887
	(98.60–116)	(100–115)		(100–115)	(100–115)	
	107	105.70		105.70	106.50	
	(105.05–110)	(105–109)		(105–109)	(103–109)	
K^+^	4.01 ± 0.63	4.04 ± 0.49	0.788	3.88 ± 0.43	4.30 ± 0.50	0.175
	(0.92–5.80)	(3.10–5.10)		(3.10–4.50)	(3.80–5.10)	
	4	4		4	4.30	
	(3.60–4.40)	(3.80–4.40)		(3.55–4.15)	(3.80–4.70)	
pH^−^	7.47 ± 0.08	7.47 ± 0.06	0.888	7.48 ± 0.07	7.46 ± 0.05	0.341
	(7.214–7.670)	(7.350–7.605)		(7.350–7.605)	(7.401–7.553)	
	7.47	7.47		7.49	7.45	
	(7.42–7.52)	(7.43–7.51)		(7.43–7.53)	(7.43–7.48)	
pCO_2_	25.79 ± 5.92	24.58 ± 5.04	0.535	23.14 ± 4.70	26.84 ± 5.03	0.063
	(13.90–43.70)	(15.80–33.10)		(15.80–32.70)	(17.80–33.10)	
	25	24.25		23.60	26.60	
	(22–29)	(22.20–27.90)		(19.10–24.80)	(24.40–30.50)	
HCO_3_^−^	20.97 ± 2.29	20.67 ± 1.80	0.431	20.32 ± 1.79	21.23 ± 1.81	0.297
	(11.70–27.40)	(17.70–24.00)		(17.70–24.00)	(18.50–23.90)	
	21.20	20.70		20.40	21.30	
	(19.50–22.50)	(19.40–21.40)		(18.60–21.40)	(19.70–22.40)	
lactate	25.14 ± 13.45	22.78 ± 9.37	0.813	23.40 ± 9.57	21.40 ± 9.84	1.000
	(9–95)	(8–49)		(15–49)	(8–32)	
	21	19.70		19.40	20	
	(16–29)	(17.50–28.50)		(18–28)	(17–30)	
